# Lateral Diffusion of Nutrients by Mammalian Herbivores in Terrestrial Ecosystems

**DOI:** 10.1371/journal.pone.0071352

**Published:** 2013-08-09

**Authors:** Adam Wolf, Christopher E. Doughty, Yadvinder Malhi

**Affiliations:** 1 Department of Ecology and Evolutionary Biology, Princeton University, Princeton, New Jersey, United States of America; 2 Environmental Change Institute, School of Geography and the Environment, University of Oxford, Oxford, United Kingdom; University of British Columbia, Canada

## Abstract

Animals translocate nutrients by consuming nutrients at one point and excreting them or dying at another location. Such lateral fluxes may be an important mechanism of nutrient supply in many ecosystems, but lack quantification and a systematic theoretical framework for their evaluation. This paper presents a mathematical framework for quantifying such fluxes in the context of mammalian herbivores. We develop an expression for lateral diffusion of a nutrient, where the diffusivity is a biologically determined parameter depending on the characteristics of mammals occupying the domain, including size-dependent phenomena such as day range, metabolic demand, food passage time, and population size. Three findings stand out: (a) Scaling law-derived estimates of diffusion parameters are comparable to estimates calculated from estimates of each coefficient gathered from primary literature. (b) The diffusion term due to transport of nutrients in dung is orders of magnitude large than the coefficient representing nutrients in bodymass. (c) The scaling coefficients show that large herbivores make a disproportionate contribution to lateral nutrient transfer. We apply the diffusion equation to a case study of Kruger National Park to estimate the conditions under which mammal-driven nutrient transport is comparable in magnitude to other (abiotic) nutrient fluxes (inputs and losses). Finally, a global analysis of mammalian herbivore transport is presented, using a comprehensive database of contemporary animal distributions. We show that continents vary greatly in terms of the importance of animal-driven nutrient fluxes, and also that perturbations to nutrient cycles are potentially quite large if threatened large herbivores are driven to extinction.

## Introduction

Nutrient availability is of primary importance in controlling the primary productivity of the biosphere. The nature of nutrient limitation is mediated between exogenous inputs and various processes taking place *in situ* that control conversion of unavailable nutrients into bioavailable forms; the accumulation of nutrients cycling between different pools; and the rate of losses from these pools [1]. Because a fraction of nutrients are inevitably leaked in any cycle, in the long-term the mean nutrient content of an ecosystem is determined by the balance between the gains and losses of nutrients from the ecosystem [2]. To the extent that exogenous nutrients are important in the nutrient budget of an ecosystem, these are often thought to arrive by abiotic processes, such as dust deposition, erosion, and runoff. These processes can be embodied in coupled ordinary differential equations [e.g. 3, 4].

This paper is an attempt to formally investigate a complementary, biotic, process that can transport nutrients into and across ecosystems: the *lateral* translocation of nutrients by mammalian herbivores, in dung or flesh. Specifically, we investigate horizontal translocation of nutrients as a diffusion process, in which the horizontal flux is proportional to a diffusion coefficient acting on a nutrient gradient. The main topics of this paper are (a) the derivation of a quantitative theoretical framework to understand lateral diffusion of nutrients by herbivores; (b) the empirical calculation of a diffusion coefficient from a compilation of field studies; (c) the analysis of a reaction-diffusion equation describing the time rate of change of phosphorus availability in a location as a function of horizontal diffusion, first order losses, and external inputs and (d) a global analysis of the magnitude of mammalian herbivore-mediated diffusion. Our goal is to understand the circumstances under which herbivore-mediated processes are dominant processes in ecosystem nutrient budgets, with special attention to the impact of global defaunation on ecosystem function. In this paper, “animal” will refer to mammalian herbivores unless otherwise specified.

There is a large body of work applying advection-diffusion equations to characterize animal movement [5]. However, there is considerably less application of such models to understanding the budgets of materials associated with animal movement, particularly nutrients ingested as biomass and excreted as urine, dung, and eventually falling as the body mass of the dead animal itself.

By contrast, there is a separate body of work focusing on animals and their impact on nutrient accumulation and the rate of nutrient cycling in ecosystems, generally on sites where animals are concentrated. The first deep investigation of this field, G.E. Hutchinson’s *Biogeochemistry of Vertebrate Excretion* [6], focused exclusively on guano deposits, that is nutrients from excreta that accumulate when large organisms feed over a “wide trophophoric field and return to a limited site for rest or reproduction.” Hutchinson’s work in many ways touches on themes that are appropriate in the present article, namely global-scale patterns of physical and biological geography; short (intra annual) timescales of behavior nested within long (Quaternary) timescales of biogeochemistry; and the behavior, diet, and population biology of the species under consideration.

As in Hutchinson [6], research on the role of large mammalian grazers in biogeochemistry has a tendency to focus on the rate of nutrient cycling and consequent productivity in regions of animal concentration [7], [8], rather than spatial linkages between nutrient source and sink regions. There are notable exceptions to this pattern, where herbivores provide nutrients to nutrient-limited regions [9]–[12].

What is the relevance for studying the role of animals in biogeochemical cycles over such long timescales? Typically, analyses indicate that herbivore-mediated nutrient fluxes are small compared with other terms in the nutrient budget [13], [14]. However, there is an increasing recognition that probably all ecosystems in the ‘Anthropocene’ are at disequilibrium following a human perturbation from some prior state [15]. While many such perturbations are obvious, such as land use change, others are subtle or indirect, such as species invasions, eutrophication, CO_2_ increase, atmospheric warming, and the like. Among these perturbations, we are interested in exploring the consequences on ecosystem function of the ongoing global defaunation event [16], which may perturb nutrient cycles far into the future in ways that are not fully understood.

The goal of this paper is to develop a theoretical framework for understanding the effects of mammal removal on lateral rates of nutrient transport. The framework is kept general, and could in principle be adapted to different nutrients (including micronutrients such as sodium or calcium) by adding details specific to the element. However, we will focus on phosphorus as the target nutrient in this work, because the timescales associated with its gain and loss terms are long [3], [4], [17].

## Methods

### Diffusion of Nutrients Arising from Animal Movement

The exchange of material between two locales is generally treated by one or both of two main processes: advection and diffusion. In advection, the flux of material in the *x* direction is equal to the concentration of the material *n* (mass/volume) times a velocity *u* (length/time), that is *J_x_* = *nu*. In diffusion, the movement from high to low density flux is negatively proportional to the local concentration gradient, −dn/dx, with the constant of proportionality termed the “diffusivity” *D* (length^2^/time): *J_x_* = −*D*(*dn/dx*). In general, a diffusivity can be derived from a random walk to characterize the aggregate statistics of a population of individually moving agents [5], [18], [19]. The dynamic equation of a probability density function governed by a random walk with length scale Δx and time scale Δt is given as [*Methods S1]*:
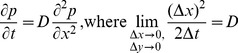
(1)


Equation [1] describes the probability density of the position of a particle, which could be an animal, a nut or disease it carries, or a particle of food in its gut. The assumptions employed in the random walk model dictate that this model does not treat long-distance migration. Additionally, we have ignored advection, i.e. bias in movement toward a particular direction, which could be used to consider a tendency to return towards a central place. We propose that there are a variety of conditions under which these assumptions may be met, on larger or smaller scales, such that the lateral transfer of nutrients by herbivores can be reasonably approximated as a diffusion-like process. At millennial scales, landscapes evolve, and migration routes, foraging hotspots, and wallows may be expected to shift location. Disturbance also serves to disrupt habitat and change vegetation type. Interannual variation in climate alters the productivity of the landscape, which drives changes in animal foraging intensity [20]. Boundaries between animal groups will change over time as internal demographics change [21]. Behavioral differences between species create differential patterns of movement. Finally, there may exist a “vasculature” in which large animals transport nutrients large distances, and progressively smaller animals diffuse nutrients more finely into the ecosystem matrix [22]. All of these phenomena suggest that the assumption of randomness may be acceptable to a first approximation, and that it is appropriate to represent the transfer of nutrients as a diffusion-like process.

It would be natural in modeling animal movement to consider Δx to be the daily displacement of a single animal (DD; km/day), where Δt is a day. The present challenge is to consider how to extend this work to predict both the distance and time traversed for a particle of plant material consumed by an animal and excreted some distance apart after some passage of time, as well as the distance and time traversed by the animal itself between consumption of plant material comprising the animal’s biomass, and its own eventual mortality. In addition, we need to extend the analysis from a single particle of plant material to the aggregate of all such transported particles, as well as consider its nutrient content.

For ingestion and excretion, the appropriate length scale in the diffusivity is the daily displacement multiplied by the average gut passage time (PT; days), and similarly the time scale for this transport would be PT. Hence, based on Equation [1], whereas the diffusivity for animal position is D_animal_ ∼ DD^2^/2 (km^2^/day), the diffusivity for transport of its excreta is D_excreta_ ∼ (DD*PT)^2^/(2*PT), where the numerator is also in km^2^ and the denominator is in days.

The diffusivity for nutrients incorporated into animal bodymass, and especially bones, D_body_, is associated with different time and length scales than for defecation. The mean residence time of a mineral in the body, e.g. phosphorus in bones, will be the mean time between apatite formation and death. As the time spent as a mature adult becomes long in relation to the time spent growing, this time scale comes to approximate its mean lifetime L (days) *[Methods S4; Figures S1 & S2]*. The length scale is linked to its home range (HR; km^2^). If the HR is interpreted to be the area that contains 95% of the probability density of an animal over its lifetime, then the root mean squared displacement would be RL = HR^0.5^/2π. An estimate of the diffusivity for bodymass is then D_body_ = RL^2^/2L = HR/(8π^2^L).

A similar equation as [1] was developed to estimate the diffusivity of a nutrient transported and redeposited by animals in excreta and biomass *[Methods S1]*. The development recognized that the mass flux of transported nutrient is determined by the population density of animals (PD; #/km^2^) that consume dry matter (DM) to fulfill their metabolic requirements (MR; kgDM/animal/day). The product of PD and MR equates to a population consumption rate of DM (denoted Q), such that QΔt is the mass of DM consumed in Δt (kg DM/km^2^). The consumption of the nutrient itself is then determined by Q(x,t) times the nutrient content of the consumed biomass ([P](x,t); kg P/kg DM), where Q[P] has units kg P/km^2^. Some fraction ε of consumed nutrient is incorporated into bodymass, while the remainder (1-ε) is excreted. Finally, a normalization is introduced, the abundance of edible biomass (αB; kg edible DM/km^2^), to represent the state variable as a mass per area. The resultant equation *[Methods S2]* is the sum of two diffusion contributions, one capturing the transport of nutrients in excreta, the other the transport in bodymass:
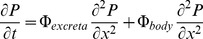
(2)where:



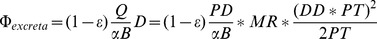
(3)

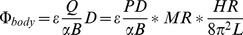
(4)


## Results and Discussion

### Diffusion Coefficients Vary as a Function of Body Size

We note that all of the terms in Φ, save the edible plant biomass αB, are known to vary systematically with herbivore body size M (kg bodymass), including some terms not considered in detail here, such as the energy content of consumed dry matter. There is a rich literature in allometric and metabolic scaling that attempts to explain these patterns, but we note here only that they exist and that we can employ them to approximate the magnitude of Φ for animals of different sizes, including animals for which we have little or no behavioral or physiological data. Whether or not every power law can be explained by fundamental theory, such power laws are useful empirical descriptors of how a particular phenomenon varies across orders of magnitude of biomass (Table 1).

**Table 1:** Allometric fits for behavioral and physiological phenomena used in the calculation of diffusivity†.

Dependent Variable	Units	Equation	n	r^2^
Population Density, *PD*	#/km^2^	87.6*M^−0.724^	366	0.71
Metabolic Demand, *MR*	kgDM/#/day	0.021*M^0.716^	131	0.96
Maximum Longevity, *L_max_*	days	4816*M^0.164^	294	0.52
Mean Longevity, *L*	days	1305*M^0.173^	170	0.57
Day Range, *DD*	km	0.453*M^0368^	113	0.52
Home Range, *HR*	km^2^	0.0416*M^1.09^	171	0.76
Range Length, (√*HR*)	km	0.204*M^0.546^	171	0.76
Passage time, *PT*	days	0.29*M^0.26^	–	–
Fecal Diffusivity‡, *Φ_exreta_*	(kgDM/km^2^) *(km^2^/day)	0.053*M^1.011^	–	–
Fecal Diffusivity†, *Φ_exreta_*	(kgDM/km^2^) *(km^2^/day)	0.050*M^1.166^	15	0.67
Body Diffusivity‡, *Φ_body_*	(kgDM/km^2^) *(km^2^/day)	8.62*10^−7^*M^0.917^	–	–
Body Diffusivity†, *Φ_body_*	(kgDM/km^2^) *(km^2^/day)	4.84 *10^−7^*M^0.897^	40	0.68

† statistical fit to primary data;

‡ estimate computed from scaling coefficients.

Data were collated from a variety of sources, and reconciled to a common taxonomic authority, Mammal Species of the World, 3^rd^ Edition [MSW3, 23] (http://www.bucknell.edu/msw3/export.asp). Data were restricted to terrestrial mammals at the species level, totaling 5278 unique taxa. Statistics were only calculated for herbivores, although other taxa with available data (insectivores, carnivores, and those having unknown diet) are plotted for reference. Data collected for this study include longevity, fecundity and metabolic rate from the AnAge database [24]; population density [25]; day range [26]; and home range [27], all of which include M as a predictor variable, as well as a dataset of M *per se* [28], which was used preferentially if available for a taxon. Passage time was not estimated from primary data, and instead the equation from Demment and Van Soest [29] was employed.

We estimated Φ as a function of M by two routes: first, we calculated the allometries for each term as a function of M (using ordinary least squares) and combined the resulting coefficients to yield an allometric equation for Φ that results from scaling arguments. Second, we multiplied values for the terms in [2] and [3] available in the primary literature to estimate Φ directly for individual species, and fit the allometric equation using the data themselves. Because the primary data comprising Φ include independent primary data, as well as allometric estimates of PR that are exact functions of M, caution is urged in interpreting the goodness of fit of Φ with M.

**Figure 1:**
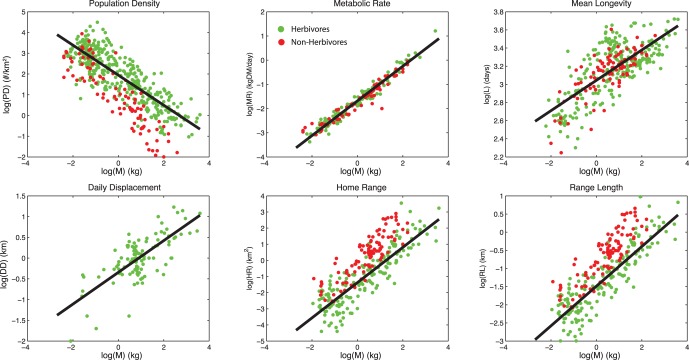
Allometric relations between bodymass M and population density, metabolic rate, mean longevity, daily displacement, home range, and range length.

**Figure 2:**
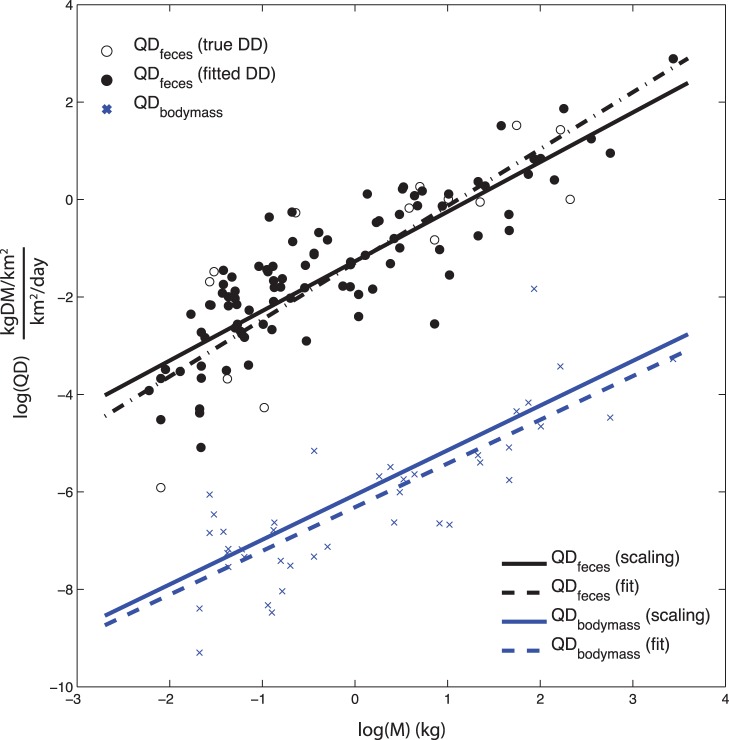
Allometric relations between bodymass M and animal-mediated nutrient diffusivity, Equations [3] and [4] in the main text. Solid lines are estimates calculated using scaling arguments, dashed lines as a fit to primary data. Circles show diffusivity by way of excreta, crosses show diffusivity by way of bodymass.

The correlations of the behavioral and physiological phenomena with M are generally strong, with r^2^≥52% (Table 1, Figure 1). The weakest correlations were found for longevity, which is in part attributable to the diverse taxonomy of the dataset: when only non-primates are considered, this correlation increases to 80% *[Figure S2 ]*. However, this term is relatively unimportant because this term only appears in Φ_body_, which was found to be 10,000x smaller than Φ_excreta_ (Table 1, Figure 2). Hence, the bodymass term can be safely neglected in nutrient flux calculations. Among terms that contribute to Φ_exreta_, the weakest correlation with M was for daily displacement. This dataset is fairly current, but nevertheless has the smallest sample size of all the presented data, suggesting that among these factors the ecology of daily movement is the least understood. The allometry of QD_excreta_ calculated using scaling arguments is nearly the same as that calculated from the primary data, suggesting there are not strong correlations between the terms that are not already accounted for by M (Table 1; Figure 2).

Both diffusivities QD_excreta_ and QD_body_ are strong functions of M (Figure 2), highlighting the importance of larger bodied species in transporting nutrients. Both diffusivities increase with body mass, and for the dominant term QD_excreta_, the scaling coefficient is >1, which shows that larger herbivores are increasingly important. Examining Equation [3], we see that large animals are important for diffusion firstly because of their large day ranges (DD^2^ ∼ M^0.736^) and secondly because of long gut passage times (PT ∼ M^0.26^). The influence of higher biomass consumption rates (MR ∼ M^0.716^) is almost exactly offset by the lower population density (PD ∼ M^−0.724^), leading to little mass dependence of biomass consumption per unit are (MR*PD ∼ M^−0.08^). This last feature reflects the “law of energy equivalence” [30], which indicates that the population-level biomass consumption should be equal across a range of M. Hence the essential role of large herbivores is embodied in the D term in Equation [3], namely daily displacement and gut residence time.

### Transport of Phosphorus in Kruger National Park

**Figure 3:**
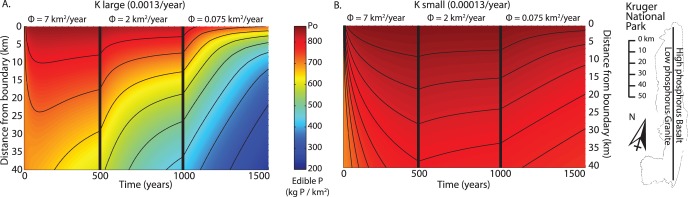
Diffusion into granitic region of KNP. Upper panel shows geometry of the simulated transect, with an inset to show the initial and boundary conditions of a transect across the substrate gradient in the absence of herbivore diffusion. Lower panels show phosphorus stocks in edible vegetation under a succession of herbivore removals, varying from Φ varies from 7 km^2^/year (estimate prior maximum) to 2 (present-day estimate) to 0.075 (estimate in the absence of herbivores >100 kg). A. P dynamics under an upper estimate of K = 0.0013/year; B. P dynamics under a lower estimate of K = 0.00013/year. Additional parameter values set to Po = 875 kg P km^−2^, G = 0.5 kg P km^−2^ year^−1^.

We next demonstrate and explore this framework with a specific case study, to test the validity of generalized allometric scaling in a specific local context. A natural locale to explore herbivore impacts on nutrient cycles is Kruger National Park (KNP), in South Africa, because of its large and well-studied animal population [31], [32] existing on a landscape with a sharp substrate gradient that impacts the phosphorus concentration and productivity of the vegetation thereon. Herbivores play a prominent role in nutrient biogeochemistry in KNP, but nevertheless a depiction of herbivores in the nutrient cycle neglects the potential for translocation by these vectors [33]. The underlying geology is that of a nutrient poor granitic landscape in the west adjacent to a nutrient rich basalt landscape in the east (Figure 3), creating sharp contrasts in nutrient concentrations in soils [34], and forages quantity and quality [35]. The geometry of this linear substrate boundary makes the park amenable to an analysis in one dimension for clarity *[Methods S3]*. The distribution of animals on the landscape is complex, having a strong component of interannual variability, as well as dietary needs and behavior preferences of wildlife [36].

In estimating the value of Φ, consider the fauna of the park. KNP has 29 mammalian herbivores greater than 10 kg (using masses from [28]). Using the masses and population densities reported in Damuth [25], the predicted biomass density per area is 18747 kg/km^2^; however the actual biomass density of the park is estimated as 3931 kg/km^2^ [37], approximately 25% of the prediction. This is in part an overestimate of the densities (e.g. elephant population density is said by Damuth to be 1/km^2^, whereas in KNP the density is ∼0.5/km^2^ [38]; moreover many species are endangered (roan tsesseble *Damaliscus lunatus*, [32]) or recovering from past defaunation (rhino, [31]). In addition, not all species occupy all areas of the park, nor overlap completely in their range [36]. For this analysis, we will analyze Φ as a potential value, as well as a current value which is ¼ of this potential.

**Figure 4:**
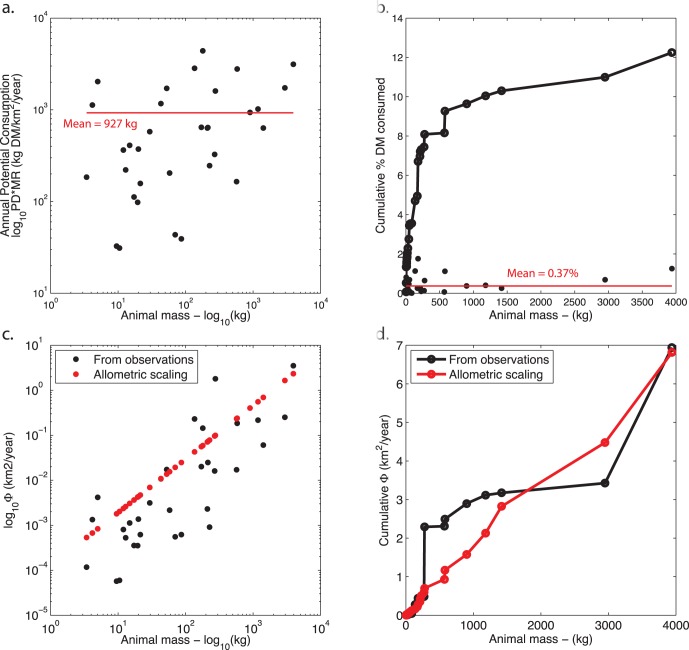
Estimates of herbivory and nutrient diffusivity in Kruger National Park by mammalian herbivores >1 kg in KNP. (a) Potential rates of consumption based on population density and metabolic demand. The mean rate of herbivory per taxon is 927 kg/km^2^ or 0.37% of the biomass standing crop. (b) cumulative rate of herbivory across body mass (c) nutrient diffusivity Φ, using observations of component terms (where possible – black points) and allometric scaling (8.672*M^1.191^; red points). (d) cumulative nutrient diffusivity Φ across body mass, based on direct observations of component terms, and allometric scaling of component terms.

The edible biomass αB is approximately 2.5 Mg/ha in KNP [35]. This figure represents only the pasture biomass; variation in this value in space and time, and additional tree foliage are not included in this figure. Furthermore the park has both grazers and browsers that select from either (or both) of these foodstuffs. However, for the purposes of simplifying this analysis, we consider that the biomass can be lumped together with the grazers and browsers consuming it. The potential consumption of each the park’s herbivores is estimated as the product of population density and metabolic demand (Figure 4a). The relation between bodymass and potential consumption is relatively flat (Figure 4a), and there are species <1 kg (e.g. the brown rat *Rattus rattus*) that have comparable rates of consumption as species >1000 kg. The mean rate of herbivory we estimate at the population level is approximately 1000 kgDM/km^2^ per taxon, regardless of size. Consequently, smaller herbivores play as important role in biomass consumption as large species – the principle of energetic equivalence [30] (Figure 4b). Collectively, we estimate that the herbivores potentially consume ∼10% of the park’s leaf biomass annually, and up to 15% if smaller herbivores are included. However, given that animal populations in KNP are less than estimated using the allometric equation, their biomass consumption is likely also less.

The consequent diffusivity Φ of this population of herbivores (excretion term only) is summarized in Figure 4c–d. Although Φ calculated from the primary data (when possible) is in general lower, there are some species that have unusually high Φ (*Loxodonta africana, Equus burchellii, Connochaetes taurinus, Damaliscus lunatus, Aepyceros melampus* from largest to smallest). Consequently, the Φ calculated using allometry is nearly the same as that calculated using primary data, approximately 7 km^2^/year. The coefficient changes little if small taxa are excluded, and even those species up to 250 kg account for just 25% of the total, which highlights the importance of the largest megaherbivores [39].

To understand the consequences of this herbivore mediated diffusion, consider a simplified budget of available phosphorus (P) governed by first order losses (K), such as runoff, and zero-order gains (G), such as dust deposition and weathering:
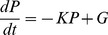
(5)


This equation is analogous to typical treatments of nutrient cycles using ODEs [4] and has an equilibrium value of G/K. The presence of herbivores adds an additional diffusive term governed by Φ and the spatial gradient in P, forming a reaction-diffusion equation:
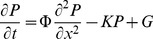
(6)


Because there is no horizontal transport mechanism in [5], the basalts and granites represent two isolated regions, each govered by their own initial conditions P_o_(x,0) and parameters K and G. However, with the presence of herbivores, there exists the possibility for P to be transported from high P to low P regions, so long as Φ >0. However, the degree to which diffusion rivals other gains and losses in the budget depends on the relative magnitudes of Φ, K, G, and the boundary condition P_o_.

The effect of herbivore diffusion from the P-rich basaltic region to the P-poor granitic region is illustrated in Figure 3 using a range of Φ for parameters G, K and P_o_ approximating that of KNP *[Methods S5]*. The numerical experiment shown simulates the P in vegetation in the granitic region following a succession of herbivore removals in 500 year intervals representing past and future defaunation. The initial condition for the domain is set to 80% the steady state value from [6], i.e. P_o_ of the basaltic region, 875 kg P km^−2^. The herbivores in the system beginning at time = 0 with the “potential” diffusivity Φ = 7 km^2^/year, followed in 500 years by an herbivore removal to represent the current “actual” diffusivity Φ = 2 km^2^/year, and finally at 1000 years the diffusivity is shown with no large herbivores (>100 kg), Φ = 0.075 km^2^/year. The analysis was run under two estimates of K, that is the larger estimate of K = 0.0013/year calculated explicitly from the mechanistic model of Buendia et al. [4] and the smaller estimate of K = 0.00013/year calculated implicitly from estimates of G [40], [41] and available P [42], under the assumption that the observed P is a steady state value including no animal inputs of P from animals, i.e. P_ss_ = G/K.

A number of features are notable from this analysis. The first observation is that the edible P under low losses (Figure 3, right panel) is improbably large, approximately double the observed value of edible P = 375 kg P km^−2^, at all levels of animal diffusion. That is, if we believe that the herbivore diffusion as outlined in this paper exists, even if only for small mammals, then the observed amount of P in edible vegetation would be expected to be considerably greater, given the rate of diffusion for even the lowest Φ within the context of the long 500,000 year timeframe of pedogenesis on these soils [43]. Because such a large value of edible P (∼750 kg P km^−2^) on the granitic soils is not observed, it would appear that the larger, explicitly calculated rate of loss is more plausible, and that the estimate of a low K as K = G/P is flawed by the assumption that Φ = 0. In other words, we argue that the system is better characterized by a higher loss rate that is compensated for by animal inputs from the basaltic substrate (Figure 3, left panel).

When K is large, the presence or absence of herbivores has strong impacts on the spatial gradients of P. In the total absence of herbivores, there is of course a sharp drop in edible P at the boundary. However, with only small herbivores (Φ = 0.075 km^2^/year), diffusion is capable of maintaining a nutrient enrichment zone above G/K up to 5–10 km away from the boundary. In the current regime with large herbivores maintained at reduced population densities (Φ = 2 km^2^/year), this zone of enrichment above G/K is extended to 20–30 km. For large herbivore densities (Φ = 7 km^2^/year), the effects of diffusion are felt throughout the granitic domain. It is clear in comparing the simulation with large K with small K that the larger the losses, the more important herbivores are in easing spatial gradients in nutrients, and conversely the more their absence is felt if they are removed. In wetter regions with higher rates of P loss, then this would imply that herbivores could play a more important role in those ecosystems in distributing nutrients.

### Global Implications

If herbivore mediated diffusion can have large effects on small scales, what is the global distribution of this phenomenon? We used the IUCN spatial database on mammal species and their ranges [44] to develop a gridded, global estimate of Φ. Although such a global gridded product should be treated with caution when applied to any specific local context, it can nevertheless provide valuable insight into broad global patterns in the capacity of animals to shift nutrients laterally within a locality. With few exceptions, each IUCN taxon was resolved to the MSW3 mammalian species list [23] and assigned a body mass from a bodymass database [28], likewise keyed to MSW3. Of the 5278 terrestrial mammals in MSW3, 2429 of these had information on body mass, largely from Smith et al. [28], although some others originated with the datasets outlined earlier. Species for which no bodymass data were available were interpolated phylogenetically, i.e. assigned to the mean value for the genus or family if necessary. Edible biomass (i.e. annual foliage production) at 1° resolution was estimated using the CASA carbon cycle model [45], summing both tree and grass/forb foliage.

**Figure 5:**
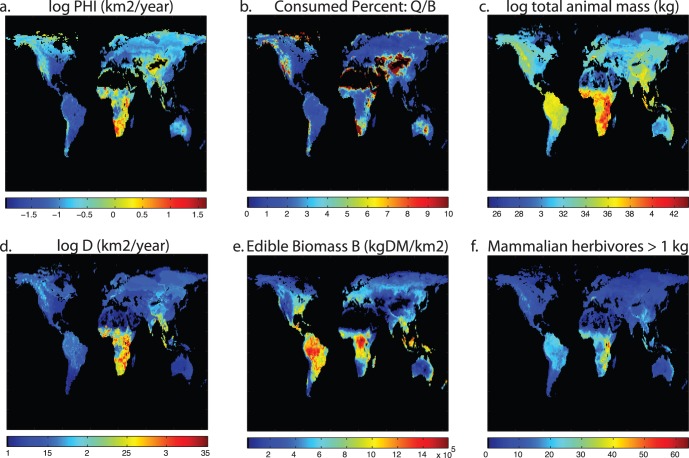
Global distribution of terms in herbivore diffusion of nutrients. (a) nutrient diffusivity Φ = DQ/αB, (b) change in Φ if all threatened species are lost.

It is apparent that there is great variation among the continents in the potential for animals to transport nutrients (Figure 5a). We note that Φ is the product of two distinct terms, namely the D term that reflects the ability of animals to transport material long distances (Figure 5d), and an herbivory term that reflects the consumption (Q) of available edible biomass (αB) (Figure 5b). Of these two terms, we noted earlier that Q varied little among species varying in M, and here Q is set to 750 kg DM/km^2^, which approximates the mean across the data presented in Figure 1. Therefore Q is more or less a function of the number of taxa present, here restricted to those >1 kg (Figure 5f). The D term, by contrast, is a strong allometric function of body size (0.0598* M^0.9962^). Predictably the Q/αB term is highest where αB is lowest, and in fact deserts (the lowest 10 percent of values here) are masked out (Figure 5b). Φ however (Figure 5a) is strongly determined by D (Figure 5d), which is greatest in Africa, particularly south and east Africa, as well as Southeast Asia and the Tibetan plateau. Africa, and to a lesser extent tropical Asia, remain the megafauna-rich continents, yet in the late Pleistocene similar high abundances of megafauna would have been found in most other continents.

The global asymmetries in Φ are striking: the Kruger example we presented earlier is at the higher end of Φ globally, with many areas reflecting a level of Φ that is most analogous to the ecosystem after all herbivores have been removed. It is not surprising that most biogeochemical research has tended to ignore this term as nearly irrelevant, for in Europe, eastern North America, and most of South America this diffusion term is 1/20^th^ or 1/100^th^ of values typical in Africa.

Naturally, the global analysis presented here omits many of the details that are known to be at play in herbivory at this scale. For one, the analysis is restricted to mammalian herbivores, which is restrictive given the importance of other clades in transporting nutrients [46], [47]. Second, we ignored relationships between herbivory and forage productivity and quality [48], instead coming up with an independent estimate that relied on IUCN species range maps and body size as predictors of biomass consumption. To the extent that species richness corresponds to productivity, our estimates are in agreement; however, this is often not the case, in particular comparing productive tropical regions such as the Amazon and Congo basins, which greatly differ in their abundance of mammals. Third, there is considerable local heterogeneity in nutrients that this global analysis ignores. This local heterogeneity in nutrients is the “potential gradient” that diffusivity acts on to create a flux, and without knowledge of this heterogeneity we can make no estimate as to the magnitude of nutrient fluxes that are borne by mammalian herbivores.

There are other aspects of local heterogeneity that deserve more careful attention as well, in particular those that impact the parameters in the model, such as population density (PD) or daily displacement (DD). We have ignored any dependence of these parameters on the underlying nutrient quality, for example the potential that high P might support higher PD or lower P might drive larger DD.

To the extent these to phenomena work in opposite directions, they might cancel each other, but nevertheless they present real challenges to the model we use and should be critically evaluated in the future. Finally, we have completely ignored other trophic levels in this analysis, particularly higher-level consumers (including humans), which would also act to limit PD.

Although these limitations are potentially important, and will shade or modify the effort to apply this work to any one place, we believe the general finding still holds. That is, that in the presence of local heterogeneity, areas with higher Φ will show a greater capacity for lateral nutrient fluxes, and that these fluxes are potentially of comparable magnitude to other major fluxes in the system.

## Conclusion

There is a rich story of the imprint of species extinctions on the global distribution of Φ (Figure 5a). It is worth considering that locales that are now considered oligotrophic, such as tropical regions like the Amazon basin, Congo basin, and southeast Asia may once have had a substantial supply of P by animal vectors despite having little renewal of surface soils by Pleistocene glaciation. In fact, as humans have gradually supplanted non-human herbivores as the major consumers of primary productivity [49], the character of P redistribution has likely also undergone a shift: whereas natural Φ probably acts like a vascular system, creating entropy by dispersing nutrients to the matrix, humans bring nutrients from the matrix and concentrate them in animal operations, much like the subjects of G.E. Hutchinson’s monograph.

**Figure 6:**
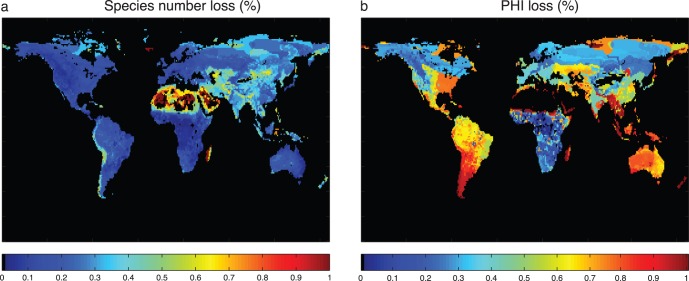
Global distribution of terms in herbivore diffusion of nutrients. (a) movement diffusivity D, (b) percent consumed biomass Q/αB, (c) total animal biomass (ie Σ mass * population), (d) nutrient diffusivity Φ = DQ/αB, (e) edible biomass αB, (f) number of mammalian herbivores >1 kg.

In summary, we have presented a mathematical framework to quantify the diffusion of nutrients by herbivores, demonstrated its applicability in the specific local context of Kruger National Park, and used these insights to mao the approximate global patterns of lateral nutrient diffusion. We propose that lateral nutrient diffusion is a previously unrecognized ecosystem service, provided by roaming large herbivores, which fuels productivity by taking nutrients from places of excess and depositing them in places of deficit. How is this ecosystem service threatened globally? A first order estimate can be obtained by exploring the consequences of extinction or movement restriction of all species identified as threatened in the IUCN redlist [44]. The fraction of species that are not extinct but currently threatened are illustrated in Figure 6a. This map highlights threats in areas that have have low intrinsic productivity (Figure 5e) and few herbivores (Figure 5f), but generally the fraction of species threatened ranges from 10–30%. By contrast, we can see in Figure 6b that extinctions to these threatened species portend large changes to Φ. This contrast indicates that the species losses are especially concentrated among taxa with high capacity to transport nutrients, i.e. large mammalian herbivores. Species extinctions historically have been felt in larger taxa [50], and in many parts of the world there do not remain many large herbivores (Figure 5f). Nevertheless, threats are felt among the remaining species, such that Φ is in many locales threatened to drop by 75–100% (Figure 6b). In addition, even if megafauna continue to persist, their population densities are greatly reduced and their ability to roam (and hence Φ) is highly constrained by habitat fragmentation and restriction to reserves. Hence the lateral flow of nutrients in wild animals is likely to be declining rapidly. It is interesting to speculate (but beyond the scope of the current study) if in many regions this loss may be compensated for by wide-ranging domesticated fauna, especially cattle and buffalos, which may play a similar but more circumscribed role in lateral nutrient diffusion.

The primary conclusion of this paper is to highlight the potential importance of lateral nutrient diffusion of nutrients by vertebrate herbivores. The framework we have developed is necessarily approximate when applied to local situations, and needs to be tested with focused empirical studies in specific ecosystems.

## Supporting Information

Methods S1 Calculating diffusivity from a random walk.
(DOCX)

Methods S2 Diffusion of nutrients transported by animals.
(DOCX)

Methods S3 Solution to 1-D PDE for diffusion away from a source region.
(DOCX)

Methods S4 Mean age of death in a population (includes Figures S1 and S2).
(DOCX)

Methods S5 Parameterization of reaction-diffusion model for Kruger National Park.
(DOCX)
