# Supplementary material for: Lateral Diffusion of Nutrients by Mammalian Herbivores in Terrestrial Ecosystems
Source: PLoS One. 2013 Aug 9;8(8):e71352. doi: 10.1371/journal.pone.0071352 (PMC3739793; doi:10.1371/journal.pone.0071352)
Supplement: Methods S1 — Calculating diffusivity from a random walk. (DOCX) [file pone.0071352.s001.docx]

## Methods S1. Calculating Diffusivity from a Random Walk

One possible derivation (Murray 2002) is as follows. Let p(x,t) be the probability that a particle leaving from x=0 at t=0 arrives at position x by time t. After a timestep of duration Δt, the particle has moved with probability α to the left or the right a distance of Δx, or remained in place with probability (1-α):


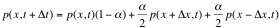
 [S1]

Rearranging terms gives:


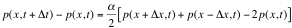
 [S2]

Consider the terms in the bracket. By Taylor expansion, the first two terms give:


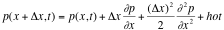
 and [S3]


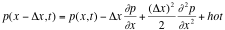
. [S4]

Discarding the higher order terms (*h.o.t.*), incorporating [S3] and [S4] into the right-hand side of [S2], and dividing both sides of the equation by Δt gives:


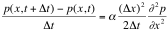
. [S5]

In the limit, as Δt and Δx are allowed to become arbitrarily small they equate to the diffusivity D, and result in the classic diffusion equation:


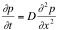
, where
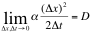
 [S6]
