# Supplementary material for: Lateral Diffusion of Nutrients by Mammalian Herbivores in Terrestrial Ecosystems
Source: PLoS One. 2013 Aug 9;8(8):e71352. doi: 10.1371/journal.pone.0071352 (PMC3739793; doi:10.1371/journal.pone.0071352)
Supplement: Methods S2 — Diffusion of nutrients transported by animals. (DOCX) [file pone.0071352.s002.docx]

## Methods S2. Diffusion of nutrients transported by animals

Next, we wish to adapt Equation 1 to consider the diffusion of a nutrient by a population of animals. Let P(x,t) represent the mass (kgP/km^2^) of the nutrient. The mass of P at position x at time t+Δt is given by:


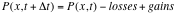
 [S7]

The *losses* term in a random walk is governed by the density of animals leaving x at time t. The loss of a nutrient in dry matter consumed and transported by a population of animals would be governed by


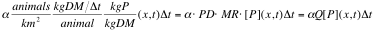
 [S8]

The loss rate of P (kgDM/km^2^) is determined by population density of animals (PD; #/km^2^) that consume dry matter (DM) to fulfill their metabolic requirements (MR; kgDM/animal/day). The product of PD and MR equates to a population consumption rate of DM (denoted Q here), such that QΔt is the mass of DM consumed in Δt (kgDM/km^2^). The consumption of the nutrient itself is then determined by Q[P](x,t), which has units kgP/km^2^, equivalent to P, the numerator on the lhs. Gains from adjacent regions will be represented analogously, as Q[P](x+Δx,t) and Q[P](x-Δx,t).

Some fraction ε of consumed nutrient is incorporated into bodymass, while the remainder (1-ε) is excreted. We may allow that some metabolism of DM also takes place, such that the concentration of the nutrient in the feces is greater than its concentration in DM; however if there are no fates of the nutrient other than bodymass or excrement, then the loss of DM by metabolism can be ignored, i.e.


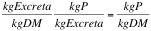
.

Consider the budget of just the fraction (1-ε) of consumed nutrient that will be excreted:


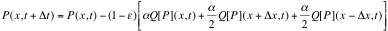
 [S9]

By analogy to the derivation for [S1], we arrive at the equation:


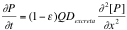
 [S10]

Adding the fraction of nutrient incorporated into bodymass we get the complete budget equation:


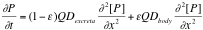
 [S11]

The state variables on the left hand and right hand sides are not the same; P is per area and [P] is per kg DM. Let B be total plant biomass (kg DM/km^2^) such that [P]B=P. We note that B has the same units as Q. If we devide both sides by B, we get:


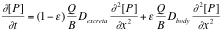
 [S12]

Equation 12 is not conceptually accurate however, because whereas B represents total plant biomass, animal consumption is only from edible parts of that biomass, say B’ = αB, where α is the edible fraction of total biomass. Likewise, only some of the P made available by feces or bodymass is allocated to edible parts. (It is assumed for simplicity here that all P made available is taken up, on a fast timescale). If these fractions can be assumed equal, then the resultant equation becomes:


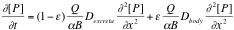
 [S13]

We have allowed that B can vary in (x,t), owing to resource gradients, and similarly, Q could vary in (x,t), by virtue of varying animal populations thriving on varying B. It is not clear at the outset whether Q(x,t)/B(x,t) can be assumed constant, because from what data is available, in drylands vegetation is thought to increase linearly with rainfall, while animal population biomass increases exponentially (East 1984, McNaughton and Georgiadis 1986). In a later section of this paper, this assumption will be examined more carefully, but for the moment if Q/B can be assumed locally constant, then:


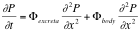
 [S14]

where the [P] terms on both sides have been multiplied by αB, and


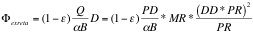
 [S15]


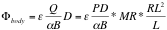
 [S16]
