# Supplementary material for: Lateral Diffusion of Nutrients by Mammalian Herbivores in Terrestrial Ecosystems
Source: PLoS One. 2013 Aug 9;8(8):e71352. doi: 10.1371/journal.pone.0071352 (PMC3739793; doi:10.1371/journal.pone.0071352)
Supplement: Methods S3 — Solution to 1-D PDE for diffusion away from a source region. (DOCX) [file pone.0071352.s003.docx]

## Methods S3. Solution to 1-D PDE for diffusion away from a source region

Consider an ordinary differential equation for a nutrient with exogenous gains G and first order losses determined by the rate constant K:


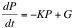
 [S17]

where G (kg P km^-2^ day^-1^) encompasses inputs such as dry or wet deposition and K (day^-1^) characterizes loss rates from leaching, runoff, erosion or volatilization. Note that for congruency with Equations 14-16, G includes only the fraction of nutrient influx that is taken up into edible biomass, and that K is likewise losses of nutrients that are potentially incorporated into edible biomass. If we are concerned with the pool immediately available for plant uptake, then these fractions are simply α. The steady state P_ss_ of this system is G/K. The addition of the diffusion term Φ adds the potential for lateral fluxes emerging from horizontal gradients in P:


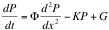
 [S18]

Two successive substititions, ie u = KP – G and v = ue^kt^, yield the homogeneous equation


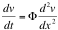
 [S19]

which is analogous to Equation 6, and has many possible solutions depending on the boundary and initial conditions. Consider the boundary condition where one edge (x=0) has a fixed concentration of the nutrient that is continuously replenished, such as a flood zone supplied by a river that overtops its banks or an eroding landscape with actively weathering substrate. Crank (1975) presented the following solution. Let a line source of material have concentration vo within a domain of width dξ, such that its initial mass is v_o_dξ. The general solution for this line source, if diffusion is only in the +x direction, is


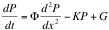
 [S20]

Integrating this expression over dξ yields:


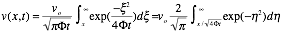
 [S21]

where η=ξ/√4Φt. In evaluating the integral, consider the error function


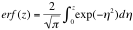
 [S22]

where erf(∞) = 1 and erf(0) = 0, and the error function complement erfc(z) = 1-erf(z). The integral then equals


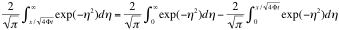
 [S23]

yielding the solution


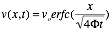
 [S24]

By the previous substitutions, v_o­_ = e^kt^(KP_o_ - G), where P_o_ is the nutrient concentration at the x=0 boundary. Backsubstituting P(x,t) = (v(x,t)e^-kt^+G)/K, the solution in conventional units is:


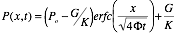
 [S25]

As t goes to infinity, P approaches the boundary condition P_o_.

The rate of propagation of P as constant values P* can be determined by rearranging the equation:


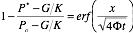
 [S26]

The inverse error function is given by the infinite series:


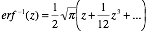
 [S27]

which yields the approximate relation


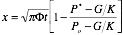
 [S28]

At the front where P*=G/K, the rate of propagation is fast, i.e. (πDt)^1/2^, and at the trailing front, where P*=P_o_, the rate is zero.
