# Supplementary material for: Lateral Diffusion of Nutrients by Mammalian Herbivores in Terrestrial Ecosystems
Source: PLoS One. 2013 Aug 9;8(8):e71352. doi: 10.1371/journal.pone.0071352 (PMC3739793; doi:10.1371/journal.pone.0071352)
Supplement: Methods S4 — Mean age of death in a population (includes Figures S1 and S2). (DOCX) [file pone.0071352.s004.docx]

## Methods S4. Mean age of death in a population

Estimation of the diffusivity associated with bodymass requires an appropriate length scale to describe the distance between consumption of a parcel of food and time of death. For the span of animal sizes under discussion, there is a spectrum in population structures that could be characterized as ranging from *r*-strategists with high birth rates and high juvenile mortality and short lifespans to *k*-strategists with lower birth rates and lower mortality and longer lifespans. We develop here a simple age-structured population model that employs simple population statistics to constrain demography, from which a mean age can be estimated.

Consider a species that reaches reproductive maturity at τmat and whose maximum age is τmax. The rate at which juveniles J become mature M is αJ = 1/τmat. The rate at which matures die is governed by μM = 4/(τmax-τmat). The factor 4 is introduced to ensure that τmax is the age by which approximately 99% of the population has passed. Mature females give birth to a litter of size L with interbirth interval I. Assuming all mature females are equivalent, and that females comprise half the mature population, the annual maternity rate is b = L/I/2. Finally, the population density is constrained to equal ρ, the number of animals per area. To achieve this population density, an additional juvenile mortality term is introduced, μJ, whose value will be estimated.

The dynamics of such a population are governed by the ODEs:


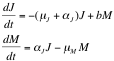


There are two corresponding expressions for the equilibria:


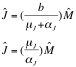


Setting these values equal determines the value for μJ:


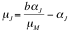


If J+M=ρ, then it can be shown that:


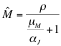


Furthermore, the fractions of the population belonging to the juvenile (fJ) and mature (fM) ages can be determined:


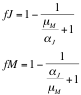


The mortality functions m(t) of the juvenile and mature populations follow the pattern

⎧ k_J_*λ_J_exp(-λ_J_*a), a<τmat

m(a) = ⎨ k_M_*λ_M_exp(-λ_M_*a), a≥τmat

⎩

where λ_J_ = μJ+αJ and λ_M_=μM and k_J_ and k_M_ are normalization factors to ensure that the integrals of each function are equal to fJ and fM, i.e.

_
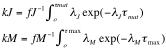
_

The mean age of death in the juvenile and mature age classes are then


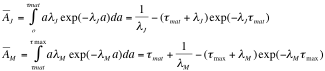


such that the mean age of death in the population overall is


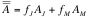
.

The AnAge database (de Magalhaes and Costa 2009) contains data on τmat, τmax, L, and I and (Damuth 1987) contains data on ρ. Together, these contain suffient data to constrain mean death age for 170 terrestrial herbivores. Figure SM1 shows the demographic rates used to constrain the model. Overall, the great fraction of the population is mature, with juveniles experiencing very large mortality rates, so the population mean longevity is strongly linked to the mean longevity in the mature population. It is clear however, that this phenomenon is size-dependent as larger species tend to have fewer offspring, with lower mortality rates, and consequently the juvenile fraction of the population is much larger by 2-3 times compared to the smaller bodied species. Primates stand out from other groups as having later time until maturity and greater maximal longevity for a given body size, as well as a lower birth rate. Consequently, their juvenile mortality rates are fairly low for a given body size.


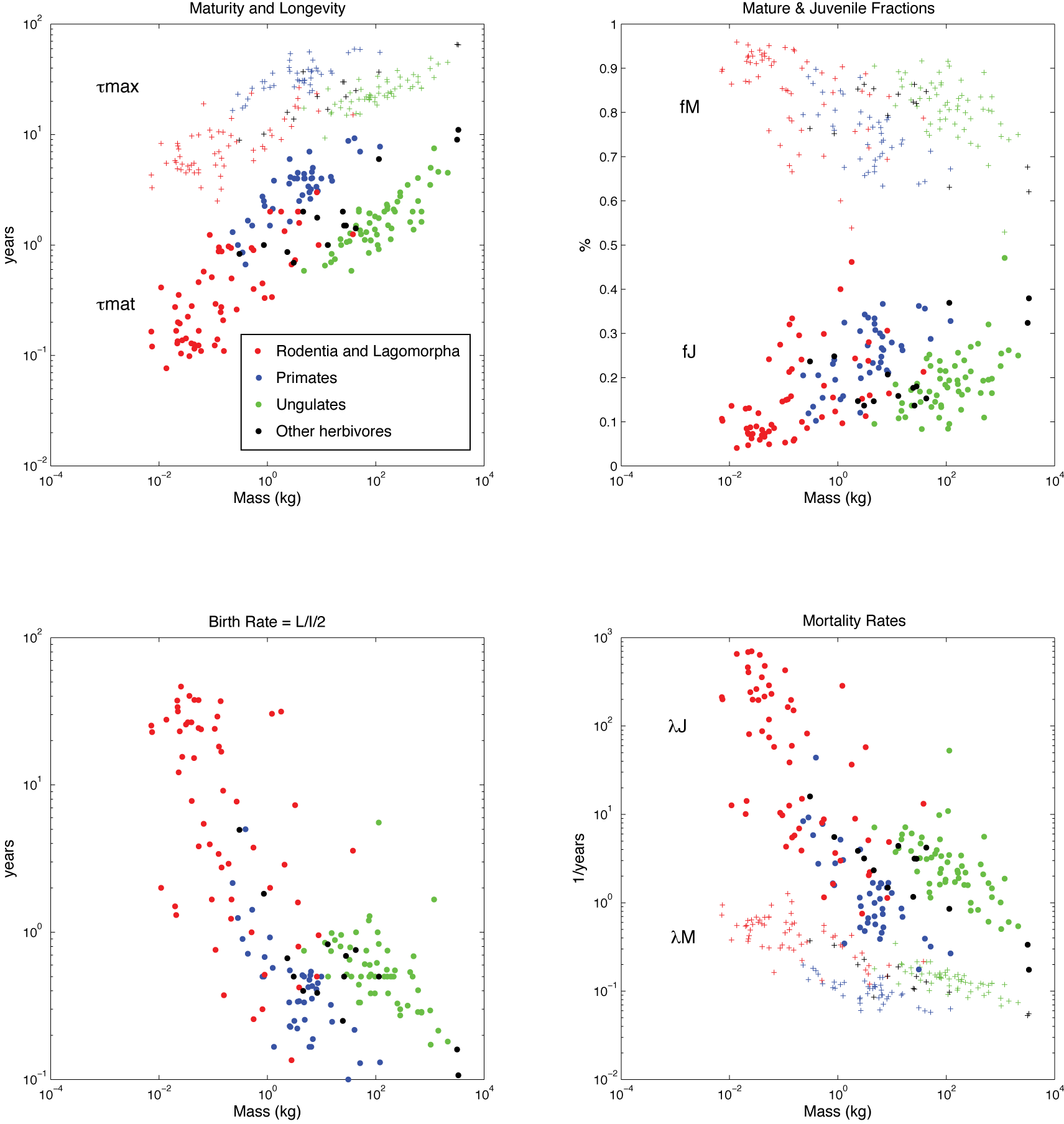


Figure S1

The estimated mean longevities (Figure S2) are approximately 20% of the maximum reported longevities (main text Figure 1). It is clear that primates fall on a different curve than the other species, with mean ages approximately double that of the others. Otherwise, the allometric constant of these mean longevities follow the same allometric scaling (0.178) as for maximum longevity (0.164).


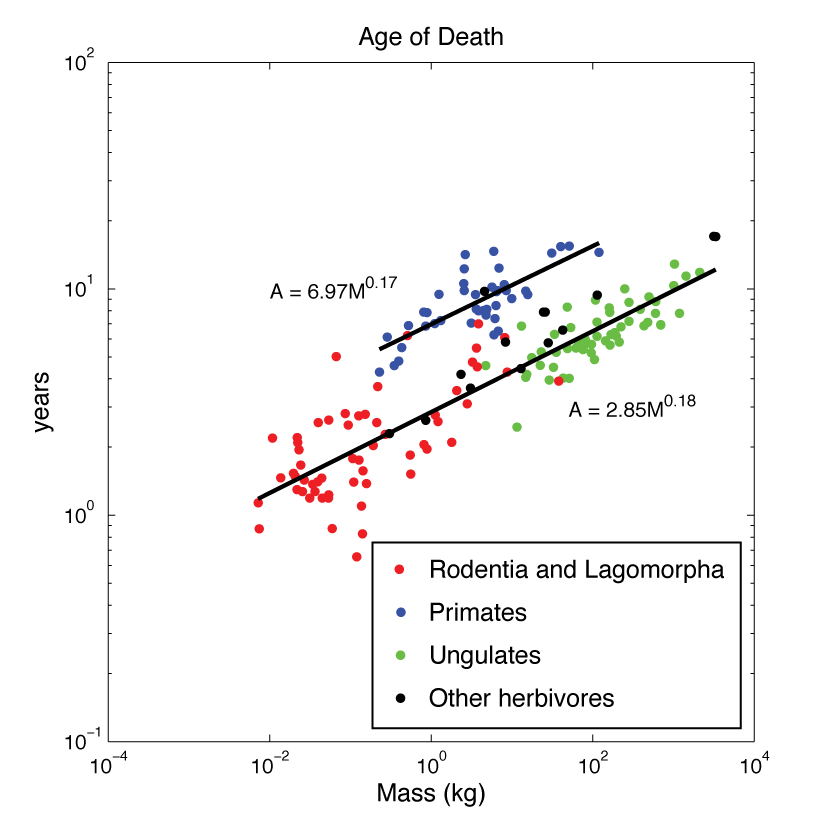


Figure S2

(damuth and deMagalhaes refs here)

Damuth, J. 1987. Interspecific Allometry of Population-Density in Mammals and Other Animals - the Independence of Body-Mass and Population Energy-Use. Biological Journal of the Linnean Society **31**:193-246.

de Magalhaes, J. P. and J. Costa. 2009. A database of vertebrate longevity records and their relation to other life-history traits. Journal of Evolutionary Biology **22**:1770-1774.
