# Supplementary material for: Lateral Diffusion of Nutrients by Mammalian Herbivores in Terrestrial Ecosystems
Source: PLoS One. 2013 Aug 9;8(8):e71352. doi: 10.1371/journal.pone.0071352 (PMC3739793; doi:10.1371/journal.pone.0071352)
Supplement: Methods S5 — Parameterization of reaction-diffusion model for Kruger National Park. (DOCX) [file pone.0071352.s005.docx]

## Methods S5. Parameterization of reaction-diffusion model for Kruger National Park

In KNP, the boundary condition is formed by the eastern basalts, which represent a source region for nutrients, because they support higher forage quantities with higher nutritive quality and nutrient content. Animals grazing on this forage that roam into the western granitic region transport with them nutrients that are then deposited in feces. Animals foraging only in the western granitic region further propagate some fraction of these nutrients further. Some of these animals of course return to the east, where there is generally a greater preference for grazing. The boundary condition for nutrients available to be transported from basalts to granites, i.e. P_o_, is the nutrient content of the edible biomass, which we assign to its nominal value of 2.5 Mg / ha, approximately the “medium productivity” value assigned by Grant et al. (2000) to pasture vegetation. Grant et al. (2000) report ~3.5 g/kg and ~14 g/kg for P and N respectively on these medium productivity swards on basaltic substrate. Higher productivity swards on basalt had 20-80% more foliar P in approximately double the standing crop of biomass, but only about 5% more foliar N, which provides evidence for a P limitation to primary productivity. These values of vegetation P concentration equate to estimates of edible P at the basalt boundary of 875 kg P / km^2^, which will define a boundary condition Po.

Now, consider the gain and loss terms governing the budget of P in the granitic substrate. The gains in P by dust in the vicinity of Kruger are estimated as 0.1 kg P km^-2^ year^-1^ by Okin et al (2004), which is at the low end of global dust deposition, with a slightly higher estimate of net P deposition by Mahowald et al (2008) of 0.1 – 0.5 kg P km^-2^ year^-1^ that includes net deposition of biomass burning P sources. Gains by weathering are comparable: Hartshorn et al. (2009) report values of 1275 kg P km^-2^ in primary mineral form^[[1]](#footnote--1).^. Based on a weathering rate of 0.0001 year^-1^ (Buendia et al. 2010), this adds 0.1275 kg P km^-2^ year^-1^ to available P in addition to dust. In sum, G is estimated to range from 0.2275 – 0.6275 kg P km^-2^ year^-1^. Losses of P from plant-available pools in KNP occur by runoff and leaching; losses by erosion are thought to be minimal on these old landscapes (Khomo 2008). Runoff and leaching is strongly dependent on soil moisture dynamics, with losses increasing exponentially once the soil is saturated (Rodríguez-Iturbe and Porporato 2004). This phenomenon was incoporated into a multi-pool model of the long-term P cycle by Buendia et al (2010) parameterized for KNP^[[2]](#footnote-0)^, which yields first-order loss rates of averaging 0.0014 year^-1^ (ranging from 0.0005 to 0.003 on dry to wet soils respectively), which is dominated by losses of dissolved P.

Hartshorn et al. (2009) report values of total and labile P in the southern granitic region of KNP of 28.07 Mg P/km^2^ and 3.8 Mg P/km^2^ respectively^[[3]](#footnote-1)^. If this value of labile P is interpreted as an equilibrium value free of lateral animal inputs, and deposition is interpreted as belonging to the labile pool in KNP (Mahowald et al. 2008), this suggests that K~=0.00013 year^-1^ under the maximum rate of deposition, i.e. 1/10^th^ that estimated by the model of Buendia et al (2010). Hartshorn et al.’s (2009) estimates of 1.5 g/kg P in leaves on granitic substrate suggest estimates of 375 kg P / km^2^ in edible biomass there , i.e. about 1/10^th^ of the labile pool or 1.35% of the total P pool, consistent with expectations (Scholes et al. 2003). For the work presented in this paper, G will be divided by 10 to represent the fraction of deposition that is manifest in edible biomass.

1. Primary mineral P = (4.5ug/g P )*(1890 Mg/m^3^ bulk density)*(15cm depth) = 1275 kg P/km^2^ [↑](#footnote-ref--1)
2. porosity = 1- ρ_bulk_/ρ_mineral_ = 0.23, where ρ_bulk_ = 1.7g/cm^3^ and ρ_mineral_ = 2.2 g/cm^3^; depth = 0.5 m; kf = 0.0005; P(organic) = 20 g P m^-2^. P(dissolved) = 0.05 g P m^-2^ [↑](#footnote-ref-0)
3. Total P: (99 ug/g total P)*(1890 Mg/m^3^ bulk density)*(15cm depth) = 28066 kg P/km^2^. Labile P (13.4 ug/g total P)*(1890 Mg/m^3^ bulk density)*(15cm depth) = 3798 kg P/km^2^. For vegetation: (2500 kg DM/ha)*(1.5ug/g veg P) = 375 kg P / km^2^. [↑](#footnote-ref-1)
